# COVID-19 vaccine hesitancy and its predictors among healthcare workers in a tertiary hospital in Ghana: A cross-sectional survey

**DOI:** 10.1371/journal.pone.0333412

**Published:** 2025-09-29

**Authors:** Israel Abebrese Sefah, Perry Ofori, Araba Hutton-Nyameaye, Peter Yamoah, Frank Baiden, Varsha Bangalee

**Affiliations:** 1 School of Pharmacy, University of Health and Allied Sciences, Ho, Volta Region, Ghana; 2 School of Public Health, University of Health and Allied Sciences, Hohoe, Volta Region, Ghana; 3 Discipline of Pharmaceutical Sciences, School of Health Sciences, University of KwaZulu-Natal, Durban, South Africa; University of Michigan School of Nursing, UNITED STATES OF AMERICA

## Abstract

**Background:**

Vaccination is one of the most effective public health interventions, but its hesitancy still poses a challenge in achieving acceptable coverage. This study was designed to assess the level of COVID-19 vaccine hesitancy and identify its influencing factors among healthcare workers in a teaching hospital in Ghana.

**Method:**

A self-administered questionnaire was used to collect data. Descriptive statistics, Chi-square test of independence, and multiple logistic regression were assessed from the data collected using Stata version 14 software.

**Results:**

A response rate of 78.55% (n = 216/275) was recorded. The majority of the participants were females (n = 135, 62.50%), aged between 20–39 years (n = 204, 94.44%), and were nurses/ midwives (n = 140, 64.81%) followed by medical doctors (n = 43, 19.91%).

COVID-19 vaccine hesitancy was 19.91% and the commonly cited reasons for this were apathy (n = 8/43, 18.60%) followed by the belief of not being at risk of infection with the virus (n = 7/43, 16.28%). Hesitancy was reduced by participant’s knowledge of the vaccine effectiveness (aOR= 0.01; CI 95%: 0.001–0.0996; p < 0.001), COVID-19 training (aOR= 0.34; CI 95%: 0.13–0.85; p = 0.022), having a past vaccination history (aOR= 0.18, CI 95%: 0.07–0.45; p < 0.001), and increased by those with the view that vaccine intake should be voluntary (aOR= 3.28; CI 95%: 1.08–10.01; p = 0.037).

**Conclusion:**

COVID-19 vaccine hesitancy was recorded among healthcare workers in this teaching hospital with apathy and belief of no risk to being infected by the virus as commonly cited reasons for this. This is a concern as healthcare workers are expected to be well-informed of the benefits and risks of vaccination. Continuous education on vaccine safety and efficacy can improve vaccine uptake among this population.

## Introduction

COVID-19 is a novel coronavirus disease that was declared a pandemic by the World Health Organization (WHO) in January 2020 [1]. Since then, the disease has had a significant impact on lives and economies worldwide [2–4]. The outbreak originated as pneumonia in Wuhan, Hubei Province of China [5]. Ghana recorded its first two cases of COVID-19 on March 12, 2020, and as at June 23, 2024, there had been a total of 775 million cases and 7 million deaths worldwide, with 172,045 cases and 1,462 deaths in Ghana [3,6]. Initial national responses included case identification, contact tracing, quarantine and isolation of infected persons, social distancing, and the widespread use of face masks and hand sanitizers [7].

Vaccination is one of the most effective interventions for combating the spread of infectious diseases in the general population [8,9]. According to the WHO, at least 70% vaccination coverage is needed to prevent transmission of SARS-CoV-2, the causative organism for COVID-19 [10], although higher thresholds have been proposed to address more transmissible variants such as Delta and Omicron [10–12].

Despite this evidence, vaccination campaigns worldwide have been constrained by vaccine hesitancy [13–15]. Vaccine hesitancy is defined by the WHO Strategic Advisory Group of Experts (SAGE) as the delay in accepting or refusing vaccination despite the availability of vaccination services [16]. It is acknowledged to be complex, context-specific, and dynamic, shaped by factors of complacency, convenience, and confidence [17,18].

Within Ghana, studies exploring COVID-19 vaccine hesitancy among healthcare workers have reported divergent findings. For example, Agyekum et al. (2021) documented a hesitancy prevalence of 60.7% among healthcare workers in Accra, a strikingly high figure at the time of early vaccine rollout [19]. Similar rates of such hesitancy have been observed among this group in Ghana and other sub-Saharan African (SSA) countries [20–24]. These further highlight the role of vaccine efficacy and safety, availability and accessibility, vaccination history, occupational status, societal and peer influence and conspiracy theories in shaping vaccine attitudes among health professionals in Ghana [19,23–27]. These discrepancies reflect differences in study timing, geographical location, cadre of health workers sampled, and the evolving information environment.

Several studies have been conducted globally to assess COVID-19 vaccine hesitancy among the general population in most countries [28–32]. Given that healthcare workers are at increased occupational risk and serve as trusted sources of health information for the wider population, understanding the factors underlying their vaccination decisions remains a critical public health priority. However, while several studies have assessed vaccine hesitancy among the general population in Ghana, relatively fewer have focused specifically on healthcare workers and particularly those in teaching hospitals [19,22–24,27,33]. The present study therefore seeks to assess the prevalence and determinants of COVID-19 vaccine hesitancy among frontline clinical staff at Ho Teaching Hospital in the Volta Region of Ghana, thereby contributing to the limited but growing body of Ghanaian evidence on this subject.

## Materials and methods

### Study site

The study was conducted in Ho Teaching Hospital (HTH), a 354-bed capacity tertiary health facility located in the Volta Regional capital, Ho in Ghana [34]. It has about 1200 clinical and non-clinical staff. The clinical departments in the hospital are Surgery, Internal Medicine, Paediatrics, Obstetrics and Gynaecology, Pharmacy, Laboratory and Radiology. The hospital is patronized by walk-ins and referred clients from the region and clients from neighbouring countries including the Republic of Togo, Benin, and the Federal Republic of Nigeria. It provides general and specialised services to about 7000 outpatients and 3000 in-patients every year [35].

### Study population

The study focused on front-line clinical healthcare workers as they are more likely to come into contact with COVID-19 patients. The targeted study participants included medical doctors, nurses, midwives, pharmacists, and laboratory personnel. The breakdown of the target group is as follows: 24 pharmacists, 94 doctors, 110 laboratory personnel, and 862 nurses and midwives.

### Sample size and sampling method

The Raosoft online sample size calculator was used to estimate the sample size. Based on a margin of error of 5%, confidence level of 95%, population size of 1090, and a hesitancy response of 60.7%, which was based on a similar previous study [19], the calculated sample size was 275.

The various targeted healthcare workers were recruited based on their calculated population proportional to their size; leading to the recruitment of 24 medical doctors, 6 pharmacists, 217 nurses and midwives and 28 laboratory personnel. The questionnaires were then distributed to them to respond to after they had consented to participate in the study.

### Data collection and analysis

The data for this study were collected with a structured self-administered questionnaire (supplementary file) that was administered both via Google online form and paper-based questionnaire. Data were collected between 1^st^ June 2023 and 30^th^ August 2023. The questionnaire was adapted from previous similar studies [19,20,23].

It had three sections which consisted of sociodemographic factors including age, sex, religion, marital status, education level and occupation; participants’ job details including type of occupation, years of practice, and role at the COVID-19 treatment at the facility; clinical characteristics and vaccine history including medical history, COVID-19 vaccine type, number of doses and vaccine acceptance/ refusal, participants’ knowledge, and perception concerning the COVID-19 vaccine, and reasons for refusal and acceptance of vaccine. The outcome variable was vaccine hesitancy, and this was defined as one’s refusal to take the vaccine in spite of its availability [16].

The data obtained from the online Google forms and hardcopy questionnaires were entered into a Microsoft Excel version 2016 sheet. The data were then cleaned and imported to Stata version 14 software (StataCorp, College Station, TX, USA) for analysis. Descriptive statistics in the form of percentages of variables, and then Chi-square test of independence were performed to assess the association between these variables and the outcome variable (status of vaccine hesitancy). Multiple logistic regression was then conducted for the variables that showed statistical significance with a p-value less than 0.05 to assess for the predictors of vaccine hesitancy.

### Ethical considerations

Ethical clearance was sought from the Ethics Committees of both the University of Health and Allied Science (UHAS-REC A.7 [46] 22–23) and Ho Teaching Hospital (HTH-REC (36) FC_2022). Written informed consents were sought from both online and in-person participants before the commencement of the study. Information on the participant information leaflet which included study details, and the benefits and risks of participating were given to each participant. Data collected were anonymized to safeguard confidentiality and kept secured with the principal investigator.

## Results

### Sociodemographic and clinical characteristics of participants

A total of 216 healthcare workers responded to the survey giving a 78.55% response rate. Majority were females (n = 135, 62.50%) and aged between 20–39 years (n = 204, 94.44%), were Christians (n = 212, 98.15%) and had never been married (n = 147, 68.06%). (Table 1).

**Table 1:** Descriptive analysis of sociodemographic and clinical characteristics of the health care workers (n = 216).

Variables	Frequency (%)
**Age (years) (n = 216)**	
20-29	128 (59.26)
30-39	76 (35.19)
40-49	9 (4.17)
50-59	3 (1.39)
**Sex (n = 216)**	
Female	135 (62.50)
Male	81 (37.50)
**Religion (n = 216)**	
African Traditional Religion	2 (0.92)
Atheist	1 (0.46)
Christian	212 (98.15)
Moslem	1 (0.46)
**Marital status (n = 216)**	
Married	67 (31.02)
Never married	147 (68.06)
Separated	2 (0.93)
**Highest attained educational level (n = 216)**	
Primary/ junior high level	15 (6.94)
Masters/PhD/Fellowship	15 (6.94)
Senior high school graduate	2 (0.93)
Undergraduate/Diploma	184 (85.19)
**Category of healthcare worker (n = 216)**	
Laboratory personnel	18 (8.33)
Medical doctors	43 (19.91)
Nurse and Midwife	140 (64.81)
Pharmacist	15 (6.94)
**Years of practice (n = 216)**	
1-10	199 (92.13)
11-20	14 (6.48)
21-30	2 (0.93)
31-40	1 (0.46)
**Member of the COVID care team (n = 216)**	
No	196 (90.74)
Yes	20 (9.26)
**Past vaccination history other than childhood vaccines (n = 216)**	
No	17 (7.87)
Yes	199 (92.13)
**Chronic disease (n = 216)**	
No	206 (95.37)
Yes	10 (4.63)
**Past medical history of COVID-19 (n = 216)**	
No	173 (80.09)
Yes	43 (19.91)
**Number of COVID diagnosis (n = 43)**	
One	34 (79.07)
Two	8 (18.60)
Three	1 (2.33)
**Family history of COVID-19 (n = 215)**	
No	184 (85.58)
Yes	31 (14.42)
**Perceived risk of getting COVID-19 in next one year (n = 216)**	
No	151 (69.91)
Yes	65 (30.09)
**Lecture on COVID-19 (n = 216)**	
No	84 (38.89)
Yes	132 (61.11)
**Contact with a COVID-19 patient (n = 216)**	
No	64 (29.63)
Yes	152 (70.37)

Nurses and midwives (n = 140, 64.81%) were the most common occupation, followed by medical doctors (n = 43, 19.91%), and undergraduate/diploma qualification n = 184, 85.19%) was commonest highest educational level. Majority of them had been practicing for less than 10 years (n = 199, 92.13%).

Majority had been previously vaccinated (n = 199, 92.13%). Most of them had no chronic diseases (n = 206, 95.37%) and had not been diagnosed with COVID-19 since the inception of the pandemic (n = 173, 80.09%). The majority of the respondents did not have a family member who had been diagnosed with COVID-19 (n = 184, 85.58%). Most of the healthcare workers had come into contact with a patient diagnosed with COVID-19 (n = 152, 70.37%). Most of them had received training on COVID-19 via a lecture mode (n = 132, 61.11%) (Table 1).

### COVID-19 vaccine hesitancy

Of the 216 participants, few of them had refused accepting the COVID-19 vaccine (i.e., hesitant) (n = 43, 19.91%). Most commonly cited reasons for vaccine hesitancy were apathy towards taking the vaccine (n = 8/43, 18.60%), followed by belief of not being at risk of infection with the virus (n = 7/43, 16.28%).

63 out of the 173 participants who delayed in taking the vaccine cited reasons of uncertainty about the long-term adverse effect of vaccine (n = 28/63, 44.44%), and poor accessibility to vaccine (n = 6/63, 9.52%). The most (n = 72/173, 41.62%) commonly cited reason for taking the COVID-19 vaccine was the perceived risk of infection from COVID-19 (41.62%) followed by workplace requirement (n = 34/173, 19.65). The commonest brand of vaccine received by most participants was ChAdOx-1s (n = 92/173, 19.08%), followed by Ad26.COV2-S (n = 33/173, 19.08%). The majority of the participants (149/173, 86.13%) who accepted vaccination received the full dose of the vaccine, and most of them (n = 111/173, 64.16%) did not receive booster doses. (Table 2)

**Table 2:** A descriptive analysis of vaccine and vaccine hesitancy.

Variables	Frequency (%)
**COVID-19 vaccine hesitancy (n = 216)**	
No	173 (80.09)
Yes	43 (19.91)
**Reason for vaccine refusal (n = 43)**	
Fear of adverse effect	2 (4.65)
Apathy towards receiving a vaccine	8 (18.60)
Belief of not being at risk of infection with the virus	7 (16.28)
Other	26 (60.47)
**Delay in vaccine acceptance (n = 173)**	
No	110 (63.89)
Yes	63 (36.11)
**Reason for delay in vaccine acceptance (n = 63)**	
Lack of trust in government and health authorities	5 (7.94)
Accessibility to vaccine barrier	6 (9.52)
Uncertainty of the long-term adverse effects of vaccine	28 (44.44)
Other	24 (38.10)
**Reason for receiving the vaccine (n = 173)**	
Other reasons	18 (10.40)
Perceived risk of Infection of COVID-19	72 (41.62)
Perceived risk of Infection of COVID-19 + Requirement at workplace	18 (10.40)
Perceived risk of Infection of COVID-19 + Requirement at workplace + Trust in the vaccine development	15 (8.67)
Perceived risk of Infection of COVID-19 + Trust in the vaccine development	16 (9.25)
Requirement at workplace	34 (19.65)
**Vaccine type (n = 173)**	
Can’t recall	16 (9.25)
Ad26.COV2-S	33 (19.08)
Other	16 (9.25)
ChAdOx-1s	92 (53.18)
BNT162b2	8 (4.62)
BNT162b2 + Ad26.COV2-S	8 (4.62)
**Adverse reaction to the vaccine after receiving (n = 173)**	
No	136 (78.61)
Yes	37 (21.39)
**Received full vaccination (n = 173)**	
No	24 (13.87)
Yes	149 (86.13)
**Received booster doses (n = 173)**	
No	111 (64.16)
Yes	62 (35.84)
**COVID-19 vaccine requirement for health workers (n = 216)**	
Mandatory	86 (39.81)
Voluntary	130 (60.19)
**Vaccination as an effective means of preventing and controlling COVID-19 (n = 216)**	
No	56 (25.93)
Yes	160 (74.07)
**COVID-19 vaccine recommendation to friends and family (n = 216)**	
No	31 (14.35)
Yes	185 (85.65)
**Change in perception on COVID-19 vaccine (n = 216)**	
No	139 (64.35)
Yes, I am now less inclined toward taking it	26 (12.04)
Yes, I am now more inclined towards taking it	51 (23.61)

Most (n = 130/216, 60.19%) responded that COVID-19 vaccines should be voluntary for healthcare workers. Furthermore, most (n = 160/216, 74.07%) of them responded that vaccination is an effective way to prevent and control COVID-19 and that they (n = 185/216, 85.65%) would advise their friends and family to take the COVID-19 vaccine. Most of them responded that their views on the COVID-19 vaccine had not changed over time (n = 139/216, 64.35%). (Table 2)

### Factors associated with COVID-19 vaccine hesitancy

Vaccine hesitancy was associated with gender status (p = 0.031), being a member of the COVID care team (p = 0.019), having received any other vaccination apart from childhood vaccines (p < 0.001), having attended COVID-19 lecture (p = 0.011), view of the vaccine requirement for health workers (p < 0.001), knowledge of the vaccine being an effective way to prevent and control COVID-19 (p < 0.001) and whether the person would recommend the COVID-19 vaccine to friends and family (p < 0.001) (Table 3).

**Table 3:** Bivariate analysis between COVID-19 vaccine hesitancy and various socio-demographic and clinical characteristics of the health care workers (n = 216).

Variables	Frequency (%)	Vaccine Hesitancy		P-value
		Yes (%) (N = 43)	No (%) (N = 173)	
Age (years) (n = 216)				0.437
20-29	128 (59.26)	23 (17.97)	105 (82.03)	
30-39	76 (35.19)	19 (25.00)	57 (75.00)	
40-49	9 (4.17)	1 (11.11)	8 (88.89)	
50-59	3 (1.39)	0 (0)	3 (100.00)	
Sex (n = 216)				0.031*
Female	135 (62.50)	33 (24.44)	102 (75.56)	
Male	81 (37.50)	10 (12.35)	71 (87.65)	
Religious status (n = 216)				0.908
African Traditional Religion	2 (0.92)	0 (0)	2 (100.00)	
Atheist	1 (0.46)	0 (0)	1 (100.00)	
Christian	212 (98.15)	43 (20.28)	169 (79.72)	
Moslem	1 (0.46)	0 (0)	1 (100.00)	
Marital status (n = 216)				0.096
Married	67 (31.02)	19 (28.36)	48 (71.64)	
Never married	147 (68.06)	24 (16.33)	123 (83.67)	
Separated	2 (0.93)	0 (0)	2 (100.00)	
Highest educational level (n = 216)				0.450
Basic Education	15 (6.94)	4 (26.67)	11 (73.33)	
Masters/PhD/Fellowship	15 (6.94)	1 (6.67)	14 (93.33)	
Senior high school graduate	2 (0.93)	0 (0)	2 (100.00)	
Undergraduate/Diploma	184 (85.19)	38 (20.65)	146 (79.35)	
Category of healthcare worker (n = 216)				0.082
Laboratory personnel	18 (8.33)	4 (22.22)	14 (77.78)	
Medical doctors	43 (19.91)	3 (6.98)	40 (93.02)	
Nurse and Midwife	140 (64.81)	31 (22.14)	109 (77.86)	
Pharmacist	15 (6.94)	5 (33.33)	10 (66.67)	
Years of practice (n = 216)				0.781
1-10	199 (92.13)	41 (20.60)	158 (79.40)	
11-20	14 (6.48)	2	12	
21-30	2 (0.93)	0 (0)	2 (100.00)	
31-40	1 (0.46)	0 (0)	1 (100.00)	
Member of the COVID care team (n = 216)				0.019*
No	196 (90.74)	43 (21.94)	153 (78.06)	
Yes	20 (9.26)	0 (0)	20 (100.00)	
Past vaccination history other than childhood vaccines (n = 216)				<0.001*
No	17 (7.87)	16 (94.12)	1 (5.88)	
Yes	199 (92.13)	27 (13.57)	172 (86.43)	
Chronic disease (n = 216)				0.422
No	206 (95.37)	42 (20.39)	164 (79.61)	
Yes	10 (4.63)	1 (10.00)	9 (90.00)	
Past medical history of COVID-19 (n = 216)				0.539
No	173 (80.09)	33 (19.08)	140 (80.92)	
Yes	43 (19.91)	10 (23.26)	33 (76.74)	
Number of COVID diagnosis (n = 43)				0.853
One	34 (79.07)	8 (23.53)	26 (76.47)	
Two	8 (18.60)	2 (25.00)	6 (75.00)	
Three	1 (2.33)	0 (0)	1 (100.00)	
Family history of COVID-19 (n = 215)				0.120
No	184 (85.58)	40 (21.74)	144 (78.26)	
Yes	31 (14.42)	3 (9.68)	28 (90.32)	
Perceived risk of getting COVID in the next year (n = 216)				0.673
No	151 (69.91)	28 (18.54)	123 (81.46)	
Yes	65 (30.09)	15 (23.08)	50 (76.92)	
Trained on COVID-19 via lecture (n = 216)				0.011*
No	84 (38.89)	24 (28.57)	60 (71.43)	
Yes	132 (61.11)	19 (14.39)	113 (85.61)	
Contact with a COVID-19 patient (n = 216)				0.923
No	64 (29.63)	13 (20.31)	51 (79.69)	
Yes	152 (70.37)	30 (19.74)	122 (80.26)	
COVID-19 vaccine requirement for health workers (n = 216)				<0.001*
Mandatory	86 (39.81)	6 (6.98)	80 (93.02)	
Voluntary	130 (60.19)	37 (28.46)	93 (71.54)	
Vaccination as an effective means of preventing and controlling COVID-19 (n = 216)				<0.001*
No	56 (25.93)	26 (46.43)	30 (53.57)	
Yes	160 (74.07)	17 (10.63)	143 (89.37)	
COVID-19 vaccine recommendation to friends and family (n = 216)				<0.001*
No	31 (14.35)	17 (54.84)	14 (45.16)	
Yes	185 (85.65)	26 (14.05)	159 (85.95)	
Change in perception on COVID-19 vaccine (n = 216)				0.066
No	139 (64.35)	30 (21.58)	109 (78.42)	
Yes, I am now less inclined toward taking it	26 (12.04)	8 (30.77)	18 (69.23)	
Yes, I am now more inclined towards taking it	51 (23.61)	5 (9.80)	46 (90.20)	

P-values with * (asterisk) means that they are statistically significant with p-value less than 0.05.

### Predictors of COVID-19 vaccine hesitancy

Vaccine hesitancy was predicted by participant’s knowledge of the vaccine being an effective means of preventing and controlling the disease (aOR= 0.01; CI 95%: 0.01–0.00; p < 0.001), COVID-19 training via lecture method (aOR= 0.34; CI 95%: 0.13–0.85; p = 0.022), their view on vaccine requirement for health workers (aOR= 3.28; CI 95%: 1.08–10.01; p = 0.037). past vaccination history (aOR= 0.18, CI 95%: 0.07–0.45; p < 0.001). (Table 4)

**Table 4:** Logistic regression between independent variables that showed statistically significant association and COVID-19 vaccine hesitancy (n = 216).

Independent Variables	aOR	95% CI	p-value
**Vaccination as an effective means of preventing and controlling COVID-19**			
No (R)	1.00		
Yes	0.18	0.07-0.45	<0.001*
**COVID-19 vaccine requirement for health workers**			
Mandatory (R)	1.00		
Voluntary	3.28	1.08-10.01	0.037*
**Member of the COVID care team**			
No (R)	1.00		
Yes	1.00		
**Lecture on COVID-19**			
No (R)	1.00		
Yes	0.34	0.13-0.85	0.022*
**Past vaccination history**			
No (R)	1.00		
Yes	0.01	0.01-0.10	<0.001*
**Sex**			
Female (R)	1.00		
Male	0.75	0.27-2.10	0.580

Where R is the reference variable, and * (asterisk) means the variable was statistically significant with p-value less than 0.05; aOR means adjusted odd ratio; C.I means confidence interval.

## Discussion

This study found a COVID-19 vaccine hesitancy prevalence of 19.91% among frontline clinical healthcare workers at Ho Teaching Hospital. When situated within the broader Ghanaian literature, this prevalence is relatively low. For instance, Agyekum et al. (2021) reported a much higher hesitancy rate of 60.7% among healthcare workers in Accra at the onset of the vaccine rollout [19]. Similarly, Lamptey et al. (2022) and Botwe et al. (2022) documented high levels of uncertainty and refusal in other Ghanaian cohorts [22,27]. In contrast, our findings align more closely with those of Alhassan et al. (2021) in Northern Ghana, who reported that a majority of healthcare workers were willing to accept vaccination, particularly when vaccines were accessible and clear communication was provided [23]. The lower hesitancy we observed may reflect the timing of our study, which was conducted when vaccines were more widely available, when healthcare workers had greater exposure to training and public campaigns, and when local experiences with COVID-19 outcomes may have reinforced the perceived importance of vaccination.

Vaccine hesitancy among healthcare workers is particularly concerning given its benefits in protecting this vulnerable population due to increased exposure and their critical role in influencing public perception and vaccine uptake [14,15,18]. The finding that hesitancy among healthcare workers was not substantially lower than in the general population in this study and others in Ghana highlights a missed opportunity to leverage healthcare workers as vaccination advocates [30,36,37]. Apathy and a self-perceived low risk of infection emerged as the most frequently cited reasons for hesitancy, agreeing with findings from studies in Saudi Arabia and France [30–32]. This persistent misperception of risk among healthcare workers is concerning, as it may undermine compliance with future vaccination campaigns and weaken broader public health interventions if left unaddressed.

This study identified several factors significantly associated with vaccine hesitancy. Female gender, not having received prior adult vaccinations, lack of COVID-19 training, belief that vaccination should be voluntary, and doubts about the vaccine’s effectiveness were all predictive of hesitancy. Gender has been identified as a predictor of vaccine hesitancy among healthcare workers [38–40]. The finding of the influence of gender on vaccine hesitancy is consistent with a previous study indicating that female healthcare workers are more likely to exhibit vaccine hesitancy, often due to fears surrounding infertility or rare side effects, such as thromboembolic events associated with the AstraZeneca vaccine [40,41]. This is particularly relevant in Ghana, where AstraZeneca (ChAdOx1-S) was the most widely administered vaccine during the initial phases of the vaccination campaign [42].

A negative past vaccination history emerged as a strong predictor of hesitancy (aOR = 0.01, p < 0.001), reaffirming findings from other settings that vaccine confidence is often built on positive historical experiences with immunization [43,44]. Similarly, healthcare workers who had undergone COVID-19 training via lecture mode demonstrated reduced hesitancy (aOR = 0.34, p = 0.022), reinforcing the value of structured educational interventions in promoting vaccine acceptance [16–18]. Another crucial determinant was the belief in the vaccine’s effectiveness to prevent and control COVID-19, which significantly lowered the likelihood of hesitancy (aOR = 0.08, p < 0.001), as was proposed by the SAGE Working Group on Vaccine Hesitancy [16]. Notably, healthcare workers who believed that vaccines should be mandatory for healthcare professionals and those who were willing to recommend vaccination to others also showed lower hesitancy, aligning with similar findings in global and African studies [20,43,44].

Despite its valuable contributions, this study has limitations. First, the use of convenience sampling may introduce selection bias and limit the generalizability of findings. Second, the cross-sectional design restricts causal inference, and the use of a single tertiary hospital as the study site may not capture broader national or regional trends. However, the study remains significant as it adds to the limited empirical evidence on COVID-19 vaccine hesitancy among healthcare workers in Ghana and highlights the need for context-specific strategies to address misinformation, build vaccine confidence, and support institutional trust.

## Conclusion

Vaccine hesitancy among healthcare workers in Ghana, while relatively low, remains a public health concern. The study found that vaccine hesitancy among the study population is primarily driven by apathy, a perceived low risk of infection, and concerns about vaccine safety. Significant predictors of hesitancy included female gender, a lack of prior adult vaccination, absence of formal COVID-19 training, and low confidence in the vaccine’s effectiveness.

Conversely, prior vaccination, training via lectures, belief in the vaccine’s protective value, and willingness to recommend it to others were associated with lower hesitancy. The predictors of vaccine hesitancy were participants’ knowledge of the vaccine effectiveness, COVID-19 training via lecture method, health workers’ view on whether the vaccine should be voluntary, and the knowledge of the effectiveness of the vaccine in preventing and controlling COVID-19.

Taken together, these findings contribute to a better understanding of COVID-19 vaccine hesitancy among healthcare workers in Ghana. They confirm that hesitancy is not static but responsive to evolving conditions such as vaccine availability and training opportunities. They also illustrate that interventions must be context-specific, as strategies that are effective in a tertiary teaching hospital may differ from those needed in rural or primary care settings.

## Recommendations

From a policy perspective, the relatively low hesitancy observed in this study suggests that structured training programs and institutional support can play a critical role in building vaccine confidence among healthcare workers. Targeted education, particularly addressing gender-specific concerns, should be prioritized. Additionally, leveraging positive vaccination histories and ensuring that healthcare workers themselves are well-informed and supported could enhance their role as advocates for vaccination in the general population. By documenting these determinants, this study not only complements earlier Ghanaian reports but also highlights pathways through which vaccine hesitancy can be effectively reduced among health professionals in similar contexts.

## Supporting information

S1 File The questionnaire used for the data collection has been added as supplementary file. (DOCX)

## References

[1] Sohrabi C, Alsafi Z, O’Neill N, Khan M, Kerwan A, Al-Jabir A, et al. World Health Organization declares global emergency: A review of the 2019 novel coronavirus (COVID-19). Int J Surg. 2020;76:71–6. doi: 10.1016/j.ijsu.2020.02.034 32112977 PMC7105032

[2] Melo-Oliveira ME, Sá-Caputo D, Bachur JA, Paineiras-Domingos LL, Sonza A, Lacerda AC, et al. Reported quality of life in countries with cases of COVID19: a systematic review. Expert Rev Respir Med. 2021;15(2):213–20. doi: 10.1080/17476348.2021.1826315 32951475

[3] Sefah IA, Essah DO, Haque M, Opanga SA, Kumar S, Chikowe I, et al. COVID-19, health care and self-medication issues in resource-limited settings: Findings and implications based on experiences in Ghana. Adv Hum Biol. 2021;11(3):224–33.

[4] Chowdhury EK, Khan II, Dhar BK. Catastrophic impact of Covid‐19 on the global stock markets and economic activities. Bus Soc Rev. 2022;127(2):437–60.

[5] Ciotti M, Ciccozzi M, Terrinoni A, Jiang WC, Wang CB, Bernardini S. The COVID-19 pandemic. Crit Rev Clin Laborat Sci. 2020;57(6):365–88. 10.1080/10408363.2020.1783198 32645276

[6] WHO. WHO COVID-19 dashboard. WHO Data. 2024.

[7] Yamey G, Schäferhoff M, Hatchett R, Pate M, Zhao F, McDade KK. Ensuring global access to COVID-19 vaccines. Lancet. 2020;395(10234):1405–6. doi: 10.1016/S0140-6736(20)30763-7 32243778 PMC7271264

[8] Ellwanger JH, Veiga ABG da, Kaminski V de L, Valverde-Villegas JM, Freitas AWQ de, Chies JAB. Control and prevention of infectious diseases from a One Health perspective. Genet Mol Biol. 2021;44(1 Suppl 1):e20200256. doi: 10.1590/1678-4685-GMB-2020-0256 33533395 PMC7856630

[9] Chen Y-T. Effect of vaccination patterns and vaccination rates on the spread and mortality of the COVID-19 pandemic. Health Policy Technol. 2023;12(1):100699. doi: 10.1016/j.hlpt.2022.100699 36415885 PMC9673057

[10] Plans-Rubió P. Percentages of Vaccination Coverage Required to Establish Herd Immunity against SARS-CoV-2. Vaccines (Basel). 2022;10(5):736. doi: 10.3390/vaccines10050736 35632492 PMC9144560

[11] Suryawanshi YN, Biswas DA. Herd Immunity to Fight Against COVID-19: A Narrative Review. Cureus. 2023;15(1):e33575. doi: 10.7759/cureus.33575 36779140 PMC9909126

[12] She J, Hou D, Chen C, Bi J, Song Y. Challenges of vaccination and herd immunity in COVID-19 and management strategies. Clin Respir J. 2022;16(11):708–16. doi: 10.1111/crj.13543 36172975 PMC9539035

[13] Hassan W, Kazmi SK, Tahir MJ, Ullah I, Royan HA, Fahriani M, et al. Global acceptance and hesitancy of COVID-19 vaccination: A narrative review. Narra J. 2021;1(3):e57. doi: 10.52225/narra.v1i3.57 38450215 PMC10914054

[14] Troiano G, Nardi A. Vaccine hesitancy in the era of COVID-19. Public Health. 2021;194:245–51. doi: 10.1016/j.puhe.2021.02.025 33965796 PMC7931735

[15] Sallam M. COVID-19 Vaccine Hesitancy Worldwide: A Concise Systematic Review of Vaccine Acceptance Rates. Vaccines (Basel). 2021;9(2):160. doi: 10.3390/vaccines9020160 33669441 PMC7920465

[16] MacDonald NE. Vaccine hesitancy: Definition, scope and determinants. Vaccine. 2015;33(34):4161–4. 25896383 10.1016/j.vaccine.2015.04.036

[17] Larson HJ, Jarrett C, Schulz WS, Chaudhuri M, Zhou Y, Dube E, et al. Measuring vaccine hesitancy: The development of a survey tool. Vaccine. 2015;33(34):4165–75. doi: 10.1016/j.vaccine.2015.04.037 25896384

[18] Kumar D, Chandra R, Mathur M, Samdariya S, Kapoor N. Vaccine hesitancy: understanding better to address better. Isr J Health Policy Res. 2016;5:2. doi: 10.1186/s13584-016-0062-y 26839681 PMC4736490

[19] Agyekum MW, Afrifa-Anane GF, Kyei-Arthur F, Addo B. Acceptability of COVID‐19 vaccination among health care workers in Ghana. Adv Public Health. 2021;2021(1):9998176. doi: 10.1155/2021/9998176

[20] Angelo AT, Alemayehu DS, Dachew AM. Health care workers intention to accept COVID-19 vaccine and associated factors in southwestern Ethiopia, 2021. PLoS One. 2021;16(9):e0257109. doi: 10.1371/journal.pone.0257109 34478470 PMC8415602

[21] Adejumo OA, Ogundele OA, Madubuko CR, Oluwafemi RO, Okoye OC, Okonkwo KC, et al. Perceptions of the COVID-19 vaccine and willingness to receive vaccination among health workers in Nigeria. Osong Public Health Res Perspect. 2021;12(4):236–43. doi: 10.24171/j.phrp.2021.0023 34289295 PMC8408417

[22] Lamptey E, Serwaa D, Appiah AB. A nationwide survey of the potential acceptance and determinants of COVID-19 vaccines in Ghana. Clin Exp Vacc Res. 2021;10(2):183–90. doi: 10.7774/cevr.2021.10.2.183 34222131 PMC8217584

[23] Alhassan RK, Aberese-Ako M, Doegah PT, Immurana M, Dalaba MA, Manyeh AK, et al. COVID-19 vaccine hesitancy among the adult population in Ghana: evidence from a pre-vaccination rollout survey. Trop Med Health. 2021;49(1):96. 34915939 10.1186/s41182-021-00357-5 PMC8674411

[24] Asumah MN, Abubakari A, Fosu B, Dzantor EK, Agyapong PD, Harrison SB, et al. Determinants of COVID-19 vaccine acceptance and hesitancy among healthcare professionals in the Kintampo North Municipality, Bono East Region, Ghana. Ghana Med J. 2022;56(3):152–9. doi: 10.4314/gmj.v56i3.4 37448993 PMC10336636

[25] Ackah M, Ameyaw L, Gazali Salifu M, Afi Asubonteng DP, Osei Yeboah C, Narkotey Annor E, et al. COVID-19 vaccine acceptance among health care workers in Africa: A systematic review and meta-analysis. PLoS One. 2022;17(5):e0268711. doi: 10.1371/journal.pone.0268711 35584110 PMC9116626

[26] Biswas N, Mustapha T, Khubchandani J, Price JH. The Nature and Extent of COVID-19 Vaccination Hesitancy in Healthcare Workers. J Community Health. 2021;46(6):1244–51. doi: 10.1007/s10900-021-00984-3 33877534 PMC8056370

[27] Botwe BO, Antwi WK, Adusei JA, Mayeden RN, Akudjedu TN, Sule SD. COVID-19 vaccine hesitancy concerns: Findings from a Ghana clinical radiography workforce survey. Radiography (Lond). 2022;28(2):537–44. doi: 10.1016/j.radi.2021.09.015 34654631 PMC8498685

[28] Alibrahim J, Awad A. COVID-19 Vaccine Hesitancy among the Public in Kuwait: A Cross-Sectional Survey. Int J Environ Res Public Health. 2021;18(16):8836. doi: 10.3390/ijerph18168836 34444585 PMC8394561

[29] Yahia AIO, Alshahrani AM, Alsulmi WGH, Alqarni MMM, Abdulrahim TKA, Heba WFH, et al. Determinants of COVID-19 vaccine acceptance and hesitancy: a cross-sectional study in Saudi Arabia. Hum Vaccin Immunother. 2021;17(11):4015–20. doi: 10.1080/21645515.2021.1950506 34353226 PMC8828146

[30] Al-Mohaithef M, Padhi BK. Determinants of COVID-19 Vaccine Acceptance in Saudi Arabia: A Web-Based National Survey. J Multidiscip Healthc. 2020;13:1657–63. doi: 10.2147/JMDH.S276771 33262600 PMC7686470

[31] Orangi S, Pinchoff J, Mwanga D, Abuya T, Hamaluba M, Warimwe G, et al. Assessing the Level and Determinants of COVID-19 Vaccine Confidence in Kenya. Vaccines (Basel). 2021;9(8):936. doi: 10.3390/vaccines9080936 34452061 PMC8402839

[32] Schwarzinger M, Watson V, Arwidson P, Alla F, Luchini S. COVID-19 vaccine hesitancy in a representative working-age population in France: a survey experiment based on vaccine characteristics. Lancet Public Health. 2021;6(4):e210–21. doi: 10.1016/S2468-2667(21)00012-8 33556325 PMC7864787

[33] Brackstone K, Atengble K, Head MG, Boateng LA. Examining drivers of covid-19 vaccine hesitancy in Ghana: the roles of political allegiance, misinformation beliefs, and sociodemographic factors. MedRxiv. 2022:2022.03.16.22272463. 10.11604/pamj.2022.43.165.37314 PMC9941608 36825126

[34] Orish VN, Akake K, Lokpo SY, Kwadzokpui PK, Amegan-Aho KH, Mac-Ankrah L, et al. Evaluating the impact of COVID-19 pandemic on complicated malaria admissions and outcomes in the paediatric Ho Teaching Hospital of the Volta Region of Ghana. PLOS Glob Public Health. 2022;2(9):e0000509. doi: 10.1371/journal.pgph.0000509 36962505 PMC10021415

[35] Lopez DM, Cervantes BYH, Emmanuel D, Agordoh PD, Almaguer FM, Lambert RG, et al. Infections of the skin among children in HO Teaching Hospital of the Volta Region, Ghana. Open Access Libr J. 2020;7(3):1–9.

[36] Acheampong T, Akorsikumah EA, Osae-Kwapong J, Khalid M, Appiah A, Amuasi JH. Examining Vaccine Hesitancy in Sub-Saharan Africa: A Survey of the Knowledge and Attitudes among Adults to Receive COVID-19 Vaccines in Ghana. Vaccines (Basel). 2021;9(8):814. doi: 10.3390/vaccines9080814 34451939 PMC8402404

[37] Cooper S, van Rooyen H, Wiysonge CS. COVID-19 vaccine hesitancy in South Africa: how can we maximize uptake of COVID-19 vaccines? Exp Rev Vacc. 2021;20(8):921–33. doi: 10.1080/14760584.2021.1949291 34252336

[38] Kose S, Mandiracioglu A, Sahin S, Kaynar T, Karbus O, Ozbel Y. Vaccine hesitancy of the COVID‐19 by health care personnel. Int J Clin Pract. 2021;75(5):e13917.

[39] Okubo R, Yoshioka T, Ohfuji S, Matsuo T, Tabuchi T. COVID-19 vaccine hesitancy and its associated factors in Japan. Vaccines. 2021;9(6):662. 34204465 10.3390/vaccines9060662 PMC8234307

[40] Cenat JM, Noorishad PG, Moshirian Farahi SMM, Darius WP, Mesbahi El Aouame A, Onesi O. Prevalence and factors related to COVID‐19 vaccine hesitancy and unwillingness in Canada: A systematic review and meta‐analysis. J Med Virol. 2023;95(1):e28156. doi: 10.1002/jmv.28156 PMC9538578 36114154

[41] Nikpour M, Sepidarkish M, Omidvar S, Firouzbakht M. Global prevalence of acceptance of COVID-19 vaccines and associated factors in pregnant women: a systematic review and meta-analysis. Exp Rev Vacc. 2022;21(6):843–51. 10.1080/14760584.2022.2053677 35285374

[42] Wise J. Covid-19: European countries suspend use of Oxford-AstraZeneca vaccine after reports of blood clots. Brit Med J. 2021. 10.1136/bmj.n699 33707182

[43] Pires C. Global Predictors of COVID-19 Vaccine Hesitancy: A Systematic Review. Vaccines (Basel). 2022;10(8):1349. doi: 10.3390/vaccines10081349 36016237 PMC9415631

[44] Fajar JK, Sallam M, Soegiarto G, Sugiri YJ, Anshory M, Wulandari L, et al. Global Prevalence and Potential Influencing Factors of COVID-19 Vaccination Hesitancy: A Meta-Analysis. Vaccines (Basel). 2022;10(8):1356. doi: 10.3390/vaccines10081356 36016242 PMC9412456

