# Supplementary material for: COVID-19 vaccine hesitancy and its predictors among healthcare workers in a tertiary hospital in Ghana: A cross-sectional survey
Source: PLoS One. 2025 Sep 29;20(9):e0333412. doi: 10.1371/journal.pone.0333412 (PMC12478964; doi:10.1371/journal.pone.0333412)
Supplement: S1 File — (DOCX) [file pone.0333412.s001.docx]

**Supplementary File: Questionnaire to Assess COVID-19 Vaccine Hesitancy among Healthcare Workers at Ho Teaching Hospital**

**Section A: Sociodemographic Factors**

1. Age (years): …………………
2. Sex

Male

Female

1. Religion

Christian

Non-Christians

1. Marital Status

Married

Never married

Separated/divorced/widowed

1. Highest Educational Level

Certificate

Diploma

First-degree

Postgraduate

**Section B: Healthcare Worker's Role at the Facility and Work Experience**

1. Category of Healthcare worker

Nurse and Midwife

Pharmacist

Medical Doctors

Allied Health workers

1. Rank in medical profession

Foundation level

Specialist

Consultant

1. How many years of practice do you have?............................................
2. Are you a member of the COVID care team?

Yes

No

**Section C: Clinical Information (Vaccine History and Hesitancy Assessment)**

1. Have you received any other vaccination apart from childhood immunization?

Yes ☐

No ☐

1. Do you have any chronic diseases?

Yes

No

1. If yes, kindly specify

Diabetes Mellitus (Type 1/ Type 2)

Hypertension

Heart Disease

Asthma

Cancer

Immunocompromised/ Immunosuppressant drug use

Smoking

Obesity BMI > 30

Not stated

1. Have you, your family member or someone you know been diagnosed with COVID-19 (Excluding your patients)?

Yes, I was diagnosed with COVID

Yes, Family Member was diagnosed with COVID-19

None of my family members were diagnosed with COVID-19

1. Was someone you personally know diagnosed with COVID-19?

Yes

No

1. Do you think you are at risk of getting COVID-19 in the next 1 year?

I believe I already have the disease and I am immune to it (Not diagnosed by a test)

No, I am confident I will not be infected

No, I already have recovered and will not get re-infected (Diagnosed by a test)

Yes, I am concerned that I will get mild symptoms, which will probably not require hospitalization

Yes, I am concerned that I will get moderate symptoms, which will probably need hospitalization

Yes, I am concerned that I will get severe symptoms, which will probably require admission to the Intensive care unit

1. Have you attended any lecture about covid-19?

Yes

No

1. Have you had contact with a covid-19 patient?

Yes

No

1. Have you received any of the COVID-19 vaccines?

Yes

No

1. Why did you receive the vaccine (skip if above answer is NO)

Perceived risk of Infection of COVID-19

Requirement at workplace

Trust in the vaccine development

1. Why have you not received the vaccine (skip if above answer is YES)

Does not believe in Vaccination

Not confident in the safety of the vaccine

Poor post Vaccine will make me sterile

Will wait until it seems safe to take the vaccine

Religious belief

1. Which type of vaccine did you receive? (You may select more one)

Pfizer

Oxford/AstraZeneca

Moderna

Janssen

Sputnik V

Sinovac

Sinopharm

1. Are you fully vaccinated? (Skip if you have not received the vaccine)

Yes (Recommended two doses)

No (One dose)

1. How many boosters have you received?

Zero

One

Two

Three

More than three

1. COVID-19 vaccine for health care workers should be:

Mandated by the employer, like the Influenza vaccine

Mandated by the government for all health care workers

Mandated by the health institution for staff

Voluntary

1. Do you think vaccination is an effective way to prevent and control COVID-19?

Yes

No

1. Would you advise friends and family to get vaccinated for COVID-19?

Yes

No
